# Demographic and Contextual Variability in the Underdiagnosis of Dementia‐Related Cardiometabolic Risk Factors in African Populations

**DOI:** 10.1002/alz70860_103747

**Published:** 2025-12-23

**Authors:** Chinedu T Udeh‐Momoh, Jasmit Shah, Litha Musili, Harrison Kaleli, Cynthia Isabel Smith, Anne Nyambura Njogu, Catherine Bikeri Onyancha, Rachel W Maina, Omonigho M Bubu, Chiadi U. Onyike, Ozioma C. Okonkwo, Rufus O Akinyemi, Zul Merali, Mansoor Saleh, Adesola Ogunniyi, Karen Blackmon, Hugh C Hendrie

**Affiliations:** ^1^ Division of Public Health Sciences, Wake Forest University, School of Medicine, Winston‐Salem, NC, USA; ^2^ Brain and Mind Institute, Aga Khan University, Nairobi, Kenya; ^3^ Aga Khan University, Nairobi, Kenya; ^4^ Brain and Mind Institute, Aga Khan University, Nairobi, Nairobi, Kenya; ^5^ NYU Grossman School of Medicine, New York, NY, USA; ^6^ Johns Hopkins University School of Medicine, Baltimore, MD, USA; ^7^ Center for Health Disparities Research, Department of Medicine, School of Medicine and Public Health (SMPH), University of Wisconsin‐Madison, Madison, WI, USA; ^8^ College of Medicine, University of Ibadan, Ibadan, Oyo, Nigeria; ^9^ Institute for Advanced Medical Research and Training, College of Medicine, University of Ibadan, Ibadan, Nigeria; ^10^ Geisel School of Medicine at Dartmouth, Hanover, NH, USA; ^11^ Indiana University School of Medicine, Indianapolis, IN, USA

## Abstract

**Background:**

Cardiometabolic risk factors (CMRFs), such as hypertension and diabetes, are modifiable contributors to dementia risk. However, these conditions are often underdiagnosed or undertreated, particularly in African populations. This study aimed to examine discrepancies between self‐reported and objectively measured CMRFs across multiple indigenous African and African diaspora cohorts, investigate sex‐specific patterns of underdiagnosis, and explore their implications for dementia risk and brain health equity.

**Methods:**

Data were analyzed from three cohorts: (1) the African American (AA) and rural Nigerian Indianapolis‐Ibadan Dementia Study cohort (*n* = 4,353; mean age 74 years; 66% female), (2) the East African (EA) **AD‐Detect Kenya project** (*n* = 51; mean age 55 years; 51% female), and (3) the EA **Brain Resilience Kenya (BRK) study** (*n* = 61; mean age 61 years; 61% female). Underdiagnosis was operationalized as self‐reported absence of hypertension or diabetes despite borderline‐to‐abnormal clinical biomarker status, using systolic blood pressure (SBP) and fasting blood glucose (FBG) levels. Sex‐specific and demographic‐related associations were assessed using logistic regression models.

**Results:**

Underdiagnosis of key dementia‐related CMRFs (hypertension and diabetes) was **widespread across all cohorts**, indicating systemic challenges in early detection and management. **Hypertension underdiagnosis** was more prevalent in **urban men** from the **East African (EA) and African American (AA) cohorts** (OR: 2.74 [0.95–7.90] and 1.65 [1.39–1.95], respectively). Conversely, **rural women** from the **Ibadan cohort** had a higher likelihood of underdiagnosed hypertension. **Diabetes underdiagnosis** was more frequent among **African American men** (OR: 1.28 [0.98–1.69]), with similar trends observed in **indigenous African men**. **Notable demographic and contextual variability** were observed, with underdiagnosis patterns differing by **sex, marital status, socio‐economic disparities and cognitive diagnosis**.

**Conclusion:**

The **systematic underdiagnosis of CMRFs** in African populations underscore **critical disparities in dementia risk identification**. These findings highlight the **urgent need for culturally and contextually adapted, gender‐sensitive, and geographically tailored prevention strategies** to improve early diagnosis and management of CMRFs. Addressing these gaps is essential for promoting **global brain health equity**, particularly for individuals of African ancestry. Future research to be presented will explore **longitudinal associations** between underdiagnosed CMRFs and cognitive decline as well as incident dementia, informing targeted interventions for dementia prevention.

